# SST-editing: *in silico* spatial transcriptomic editing at single-cell resolution

**DOI:** 10.1093/bioinformatics/btae077

**Published:** 2024-02-10

**Authors:** Jiqing Wu, Viktor H Koelzer

**Affiliations:** Department of Pathology and Molecular Pathology, Computational and Translational Pathology Laboratory (CTP), University Hospital of Zurich, University of Zurich, Zurich, Switzerland; Department of Pathology and Molecular Pathology, Computational and Translational Pathology Laboratory (CTP), University Hospital of Zurich, University of Zurich, Zurich, Switzerland

## Abstract

**Motivation:**

Generative Adversarial Nets (GAN) achieve impressive performance for text-guided editing of natural images. However, a comparable utility of GAN remains understudied for spatial transcriptomics (ST) technologies with matched gene expression and biomedical image data.

**Results:**

We propose *In* ***S**ilico* **S**patial **T**ranscriptomic **e**diting that enables gene expression-guided editing of immunofluorescence images. Using cell-level spatial transcriptomics data extracted from normal and tumor tissue slides, we train the approach under the framework of GAN (Inversion). To simulate cellular state transitions, we then feed edited gene expression levels to trained models. Compared to normal cellular images (ground truth), we successfully model the transition from tumor to normal tissue samples, as measured with quantifiable and interpretable cellular features.

**Availability and implementation:**

https://github.com/CTPLab/SST-editing.

## 1 Introduction

Recently, Generative Adversarial Nets (GAN) (Goodfellow *et al.* 2014) and GAN Inversion (Xia *et al.* 2022) have achieved remarkable editing effects on generated (Kang *et al.* 2023) and real natural images (Patashnik *et al.* 2021). These methods mostly leverage encoded textual descriptions for desired image alterations. Typically, a pre-trained text encoder (Radford *et al.* 2021) is used to generate textual representations, which are then fed into the GAN (Inversion) model for text-guided editing of natural images.

In application to biomedicine, the utility of GAN holds great promise for *in silico* modeling of disease states. Taking bulk RNA expression as the input, a recent study (Carrillo-Perez *et al.* 2023) introduced RNA-GAN that showcased the generation of tissue image tiles. Further, GAN (Inversion)-enabled editing could aid the understanding of complex biological systems and enable low-cost and efficient cellular image manipulations. Trained with (immuno)fluorescence (IF) images that capture diverse cellular states, prior studies (Lamiable *et al.* 2023, Wu and Koelzer 2023) have demonstrated the editability of GAN and GAN Inversion on cellular images.

Without molecular data at fine granularity, the above GAN (Inversion)-based approaches are nonetheless unable to perform “text-guided” editing of biomedical images. Leveraging cutting-edge spatial transcriptomic (ST) (Moses and Pachter 2022) data, we propose In **S**ilico **S**patial **T**ranscriptomic **e**diting (SST-editing) using GAN (Inversion). We first train the approach with paired cell-level ST data, e.g. mRNA transcripts as surrogates for the expression of specific genes, and IF biomarkers. By feeding edited gene expression data to the trained models, we shift tumor cell phenotypes toward the normal cell state and study the resulting editing effects on histology IF images using quantifiable and interpretable cellular features.

## 2 Materials and methods

### 2.1 Training data

Experiments are performed on the CosMx (He *et al.* 2022) hepatocellular carcinoma (HCC, liver tumor) dataset including both normal and tumor tissue slides. With a gigapixel spatial resolution, CosMx comprehensively documents a sparse 3D array of 1000-plex gene expression and associated multi-channel IF images (e.g. CD298/B2M, DAPI). In the appendix, we also report experiments on the Xenium (Janesick *et al.* 2022) lung tumor dataset.

#### 2.1.1 Cellular expression

For the fine-grained analysis at single-cell resolution, the 1000-plex gene expression is represented as a 3D sparse array center-cropped on a detected cell. After summing the 3D array along spatial dimensions, we use the 1D table of gene expression data as the “text” input for training. To study editing effects, we algorithmically edit both the complete gene panel and the expression level of selected genes including *HLA-A* and *B2M*. This is motivated by the positive correlation between the expression level of these two genes and CD298/B2M protein expression as measured by IF in the samples under study (please see also Supplementary Fig. S2).

#### 2.1.2 Cellular image

Given the striking difference demonstrated by the expression level (pixel intensity) of CD298/B2M and cellular morphology (DAPI) between normal and tumor cells, we take the combination of these two markers as the “image” modality. Consequently, the two-channel image is a 3D array center-cropped on the same cell with paired gene expression input.

#### 2.1.3 Cell subtype

**Figure 1.**
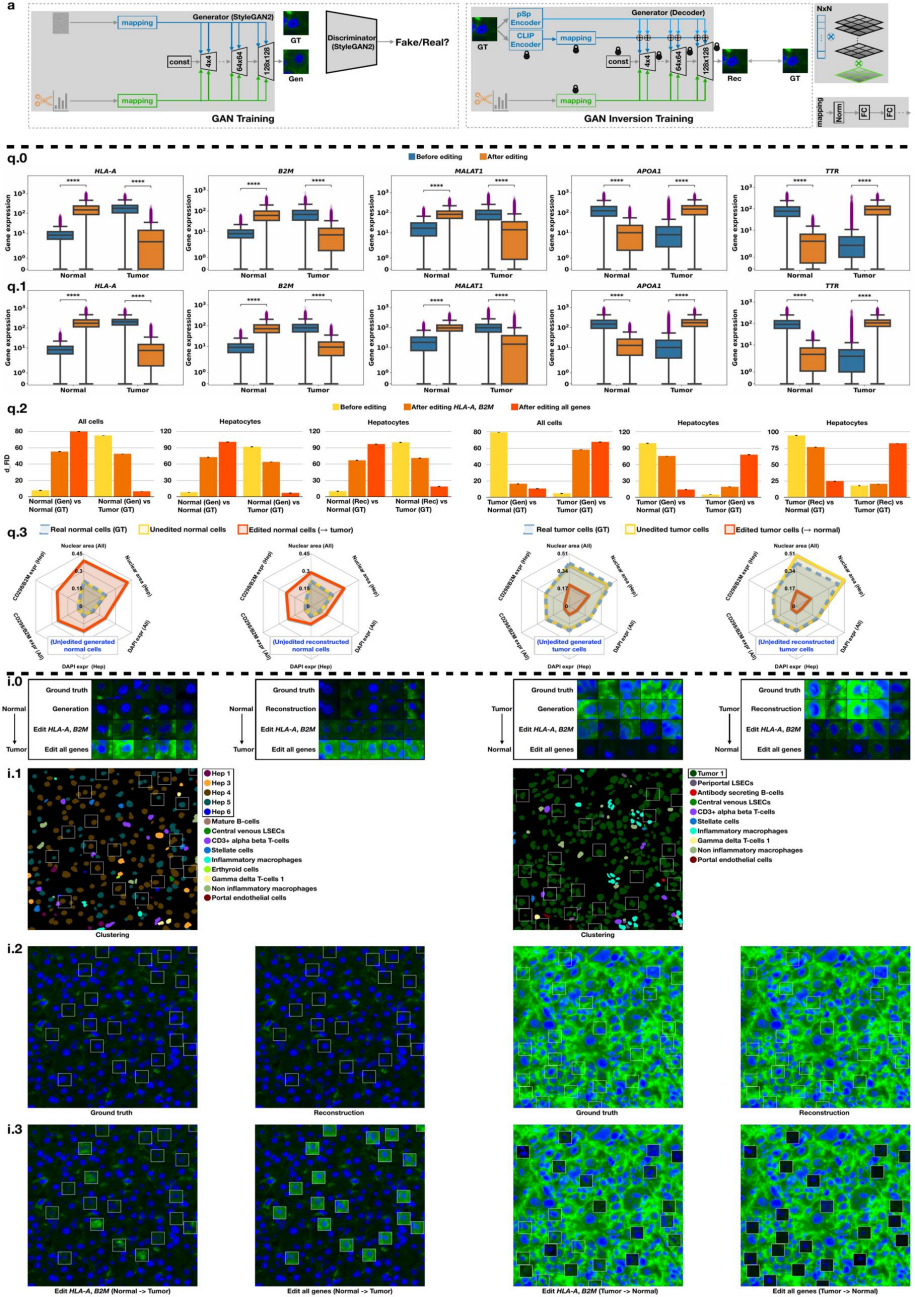
(a) Model illustrations. (q) Numerical quantification of CosMx results. (q.0 and q.1) The comparison of gene expression shifts on all the cells (q.0) and the “Hepatocyte” (Hep) subtypes (q.1) of the normal and tumor slide. Here, **** means *P* ≤.0001. (q.2) For the generated (Gen, GAN) and reconstructed (Rec, GAN Inversion) cells, the $d_{\mathrm{FID}}$ comparison of cellular state transitions *w.r.t*. all cells and Hep subtypes. We randomly repeat the $d_{\mathrm{FID}}$ computation four times and report the mean and standard deviation. (q.3) The editing effect comparison of interpretable cellular features for all the cells and hepatocytes (Hep). (i) Visual interpretation of CosMx results. (i.0) The image gallery of cellular state transitions for Gen and Rec cells. Here, we present the transition that occurred in the DAPI (blue) and CD298/B2M (green) channels. (i.1) The visualization of cell subtypes on a region of interest extracted from the normal and tumor slide. (i.2) The randomly sampled ground truth and reconstructed cellular images within the bounding boxes. (i.3) The morphological transitions of these cellular images guided by edited gene expression levels

Apart from utilizing the entire cell population, we run an ablative investigation on cell subtypes of interest. Based on the clustering annotation provided in the dataset, we select normal cells [Hep 1, 3, 4, 5, 6 in Fig. 1i.1 (left)] and HCC cells [Tumor 1 in Fig. 1i.1 (right)] to analyze the editing effects guided by expert pathologist review.

### 2.2 SST-editing

In reference to normal (or tumor) cellular images as the ground truth, we model the transition from tumor cellular images to normal ones (or vice versa) by SST-editing. To this end, we develop our approach upon the state-of-the-art StyleGAN2 (Karras *et al.* 2020) architecture.

#### 2.2.1 Step 1 (GAN training)

Instead of using convoluted latent (textual) representations for natural image manipulation, we propose to directly utilize the gene expression data for cellular image manipulation. Concretely, this is done by feeding the gene expression table to a mapping network [Fig. 1a (left)] for controlling cellular features. Taking the discriminator *D* as the adversary, we train the generator *G* to output high-quality IF cellular images. To learn distinctive features of normal and tumor cells, both networks are trained with the adversarial loss $L_{\mathrm{adc}}$ conditioned on binary cell labels [normal (0) and tumor (1)]. Together with the $R_{1}$ regulation $L_{R_{1}}$ and path length regulation loss $L_{\mathrm{path}}$ (Karras *et al.* 2020), we have the objective:

(1) $$\underset{G}{\min}((\underset{D}{\max}L_{\mathrm{adc}})\;+\;\lambda_{R_{1}}L_{R_{1}}\;+\;\lambda_{\mathrm{path}}L_{\mathrm{path}}),$$

where $\lambda_{R_{1}}$ and $\lambda_{\mathrm{path}}$ are hyperparameters. To manipulate **generated (Gen)** cellular images, we then feed edited gene expression data to the GAN model.

#### 2.2.2 Step 2 (GAN Inversion training)

Going beyond generated cellular images, we extend the analysis of editing effects to real (reconstructed) cellular images. This is carried out by GAN Inversion (auto-encoder), in which we reuse the trained generator *G* as the decoder and aim to faithfully reconstruct the input cellular image. To strike a balance between editability and reconstruction quality, we address this dual challenge by using a pre-trained lightweight CLIP (Shariatnia 2021) encoder $E_{c}$ and a “pixel2style2pixel” (Richardson *et al.* 2021) encoder $E_{p}$ [Fig. 1a (right)], respectively. Then, we apply the contrastive $L_{\mathrm{moco}}$, $l_{2}$ reconstruction $L_{2}$ and perceptually learned $L_{\mathrm{PIP}}$ loss suggested in (Alaluf *et al.* 2021), while the ID similarity loss used in the same study tailored for facial images is excluded. As a whole, we have the objective:

(2) $$\underset{E_{c},E_{p}}{\min}(\lambda_{1}L_{\mathrm{moco}}\;+\;\lambda_{2}L_{2}\;+\;\lambda_{3}L_{\mathrm{PIP}}),$$

where $\lambda_{1,2,3}$ are the hyperparameters. After training with paired gene expression data and real cellular images, we feed the edited gene expression data to manipulate the **reconstructed (Rec)** cellular images.

GAN and GAN Inversion training were run for 800 k iterations with a batch size of 16 for GAN and 8 for GAN Inversion, respectively.

#### 2.2.3 Step 3 (Gene expression-guided editing)

Due to the lack of one-on-one correspondence between individual cells on the normal and tumor slides, clear guidance for individually editing the gene expression of each cell is not available in the experimental setup. Therefore, we propose to collectively edit the gene expression of paired cells by matching the gene data distribution [i.e. sample covariance matrix (SCM) (Wu and Koelzer 2022)] of one cell population to another. We achieved this by scaling the eigenvalues and rotating the eigenbasis of SCM. For i=0,1, consider the SCM $\frac{1}{n_{i}}G_{i}^{T}G_{i}=O_{i}\lambda_{i}O_{i}^{T}$, where $G_{i}$ is the collection of $n_{i}\;p$-plex gene expression from the normal or tumor slide, $O_{i}$ is the p×p eigenbasis and $\lambda_{i}$ is the p×p (sorted) diagonal eigenvalues obtained by eigenvalue decomposition. For the collection of $G_{i}$, we apply the linear transformation $G_{i}^{\prime }=G_{i}O_{i}(\sqrt{\lambda_{(i+1)\%2}}/\sqrt{\lambda_{i}})O_{(i+1)\%2}$ such that for the edited gene collection $G_{i}^{\prime }$ it holds $\frac{1}{n_{i}}G_{i}^{\prime T}G_{i}^{\prime }=\frac{1}{n_{(i+1)\%2}}G_{(i+1)\%2}^{T}G_{(i+1)\%2}$. Due to the computational fluctuation of smaller eigenvalues and the dominant effect of the leading eigenvalue, it suffices to scale the largest eigenvalue in our experiments. By keeping the selected group of gene expression values unchanged during the linear transformation, we narrow down the editing process to the gene of interest.

For all implementation details including, but not limited to, the above key algorithmic steps, we refer interested readers to the curated GitHub repository https://github.com/CTPLab/SST-editing.

## 3 Results

Overall, we achieve three levels of experimental settings that correspond to eight conditions: Analysis of (i) generated (GAN) and reconstructed (GAN Inversion) cellular images; (ii) (selected) gene expression values before and after in silico editing; (iii) the total cell population and clinically relevant cell subtypes (“Hepatocytes”).

### 3.1 GAN (Inversion) evaluation

We determine the optimal GAN model using Fréchet Inception Distance ($d_{\mathrm{FID}}$) (Heusel *et al.* 2017), which effectively measures the statistical distance between the feature distribution of cellular image collections. Further, we report Peak Signal-to-Noise Ratio and Structural Similarity Index Measure to benchmark the GAN Inversion model. As such, the best models are determined at 550 k (GAN, Supplementary Fig. S4) and 700 k (GAN Inversion, Supplementary Fig. S5) iterations, see Fig. 1i.0, i.2 for more image visualizations.

### 3.2 Editing effect evaluation

#### 3.2.1 Cellular quantification

Before editing: blue plots of Fig. 1q.0, q.1 illustrate two groups of genes with the highest expression difference between normal hepatocytes and HCC, e.g. genes with low expression in normal and upregulation in HCC (*HLA-A*, *B2M*, *MALAT1*) and genes downregulated in HCC compared to normal (*APOA1*, *TTR*). After editing: by shifting the gene expression distribution from one cellular state to another, we witness a meaningful change in the expression of edited genes when comparing paired populations. The quantitative comparison presents a highly significant shift in normal (or tumor) gene expression patterns toward the tumor (or normal) spectrum for the total cell population (Fig. 1q.0) and hepatocyte subtypes (Fig. 1q.1, ∼80% of the total cells). After feeding the edited gene expression profiles to the GAN (Inversion) model, we measure the manipulation effects on tumor (or normal) cellular images in comparison to ground truth normal (or tumor) cells, respectively. To support the analysis of generated (Gen) and reconstructed (Rec) cellular images with interpretable morphometric features, we perform the quantification using $d_{\mathrm{FID}}$ and cellular features such as nuclear area and CD298/B2M expression level.

#### 3.2.2 Cellular interpretation

When decreasing the leading and highly overexpressed genes *HLA-A* in tumor cells and *B2M* for the CosMx HCC dataset, we model the quantifiable transition of tumor cells (large nuclei, cellular atypia, variation in nuclear size) toward normal cells. Even more striking morphological effects become evident when editing the expression of all genes, in terms of clearly decreasing $d_{\mathrm{FID}}$ scores (Fig. 1q.2), decreasing average *B2M* marker expression and reduced average nuclear area (Fig. 1q.3) as derived from the CellPose (Stringer *et al.* 2021) method. Complementary to these quantification results, normal liver cells acquire a remarkably malignant appearance (Fig. 1i.0, i.3) when driven by edited genes of interest (increase in nuclear area and overexpression of *B2M*, Fig. 1q.3) toward the tumor spectrum. Importantly, the in silico editing of *HLA-A* and *B2M* indeed correlates to the emergence or disappearance of CD298/B2M protein expression as captured by the IF imaging (green channel), supporting the reliability of biological interpretations by the proposed approach.

## 4 Conclusions

In the biomedical context, this study sheds light on the algorithmic editability of ST data. By editing tumor gene expression profiles toward the normal spectrum, we achieved the reversal of tumor cellular images to normal ones, as measured with quantifiable and interpretable cellular features. The SST-editing approach, which exhibits low ethical, legal and regulatory risks in the simulated intervention of human biological material, thus provides a new perspective to model pathological processes in real-life clinical tissue samples.

## Supplementary Material

btae077_Supplementary_Data

## Data Availability

The CosMx platform provides two comprehensive spatial arrays of 1000-plex gene expression [∼0.2 billion gene expression counts for the normal liver slide and 0.5 billion for the hepatocellular carcinoma (HCC) slide], which correspond to 340 and 464 k cells, respectively (https://nanostring.com/wp-content/uploads/2023/01/LiverPublicDataRelease.html). Similarly, the Xenium dataset offers two large-scale spatial expression maps of 392-plex predesigned and custom target genes [∼24 million and 67 million total counts for the healthy lung slide and invasive adenocarcinoma (IAC) slide individually], along with 300 k (https://cf.10xgenomics.com/samples/xenium/1.3.0/Xenium_Preview_Human_Non_diseased_Lung_With_Add_on_FFPE/Xenium_Preview_Human_Non_diseased_Lung_With_Add_on_FFPE_analysis_summary.html) and 530 k (https://cf.10xgenomics.com/samples/xenium/1.3.0/Xenium_Preview_Human_Lung_Cancer_With_Add_on_2_FFPE/Xenium_Preview_Human_Lung_Cancer_With_Add_on_2_FFPE_analysis_summary.html) cells that are detected from the two slides. CosMx: The multiplexed fluorescence imaging (MFI) data of healthy liver tissue are available via https://smi-public.objects.liquidweb.services/NormalLiverFiles.zip, the MFI data of cancer liver tissue are available via https://smi-public.objects.liquidweb.services/LiverCancerFiles.zip, the ST data are available via https://smi-public.objects.liquidweb.services/LiverDataReleaseTileDB.zip. Xenium: The ST data and whole slide image (WSI) of healthy lung tissue are available via https://cf.10xgenomics.com/samples/xenium/1.3.0/Xenium_Preview_Human_Non_diseased_Lung_With_Add_on_FFPE/Xenium_Preview_Human_Non_diseased_Lung_With_Add_on_FFPE_outs.zip, the ST data and WSI of cancer lung tissue are available via https://s3-us-west-2.amazonaws.com/10x.files/samples/xenium/1.3.0/Xenium_Preview_Human_Lung_Cancer_With_Add_on_2_FFPE/Xenium_Preview_Human_Lung_Cancer_With_Add_on_2_FFPE_outs.zip. All the above links are free and publicly available to users, without further login or registration requirements.
