# Supplementary material for: SST-editing: in silico spatial transcriptomic editing at single-cell resolution
Source: Bioinformatics. 2024 Feb 10;40(3):btae077. doi: 10.1093/bioinformatics/btae077 (PMC10914437; doi:10.1093/bioinformatics/btae077)
Supplement: btae077_Supplementary_Data [file btae077_supplementary_data.pdf]

Journal Title Here, 2022, 1–3
doi: DOI HERE
Advance Access Publication Date: Day Month Year
Paper

SST-editing: *in silico* spatial transcriptomic editing at single-cell resolution

Jiqing Wu[1,*] and Viktor H. Koelzer[1,*]

[1] Department of Pathology and Molecular Pathology, Computational and Translational Pathology Laboratory (CTP), University Hospital of Zurich, University of Zurich, Zurich, Switzerland

*Corresponding authors. Jiqing.Wu@usz.ch, Viktor.Koelzer@usz.ch

Abstract

Motivation: Generative Adversarial Nets (GAN) achieve impressive performance for text-guided editing of natural images. However, a comparable utility of GAN remains understudied for spatial transcriptomics (ST) technologies with matched gene expression and biomedical image data.
Results: We propose *In* ***S****ilico* **S**patial **T**ranscriptomic **e**diting that enables gene expression-guided editing of immunofluorescence images. Using cell-level ST data extracted from normal and tumor tissue slides, we train the approach under the framework of GAN (Inversion). To simulate cellular state transitions, we then feed edited gene expression levels to trained models. Compared to normal cellular images (ground truth), we successfully model the transition from tumor to normal tissue samples, as measured with quantifiable and interpretable cellular features.
Availability and implementation: `https://github.com/CTPLab/SST-editing`.

Xenium Dataset

We report experimental results on the 10x Xenium invasive adenocarcinoma (IAV, lung tumor) (Janesick et al., 2022) dataset including both normal and tumor tissue slides. With a gigapixel resolution, Xenium extensively curates a sparse 3D array of 392-plex gene expression and the matched DAPI image.
Cellular expression: Similar to CosMx, we utilize the gene expression table of detected cells as the 'text' input. Such a 1D table is obtained by summing the 3D sparse array of 392-plex gene expression over spatial dimensions. Moreover, not only do we examine the whole list of 392 genes but also narrow down to leading heterogeneous genes such as *MUC1*, *KRT7*, *RBM3* and *EPCAM* for a more detailed analysis of editing effects.
Cellular image: Given the 1D table of gene expression, we then center-crop the associated DAPI image on the same cell. As such, we have the paired cellular gene expression and DAPI image data for training.
Cell subtype: Differing from CosMx, the annotation of cell subtypes is not available in the Xenium dataset. Therefore, we carefully evaluated DAPI images and cell-level clustering results supplied in the raw data. We found the second cluster of the 'kmeans_2_clusters' category to be highly enriched in cells of epithelial lineage for both normal and tumor slides (Fig. 3). Given the susceptible property of epithelial cells in tumor development, we select normal lung epithelial cells and adenocarcinoma (tumor cells) identified in 'kmeans_2_clusters' for the follow-up analysis (Fig. 1 (i.1)).

Here, we run Xenium experiments using the SST-editing approach. Except for different image dimensions, *i.e.*, CosMx (2 channels, 96×96) VS Xenium (1 channel, 160×160), which are based on the feasibility of single-cell analysis and the pathologist review, the GAN (Inversion) training and in silico editing are performed under the same configurations for both datasets.

Xenium Results

GAN (Inversion) evaluation

Same as the CosMx experiments, we use the d_{FID} (GAN), PSNR and SSIM (GAN Inversion) to benchmark the model performance. As a result, the best models are obtained at 800k (GAN, Fig. 4 (c, d)) and 700k (GAN Inversion, Fig. 5 (c, d)) iterations respectively. Please see also Fig. 1 (i.0, i.2) for more generated and reconstructed image visualization.

Editing effect evaluation

Cellular quantification:(Before editing) As illustrated in blue plots of Fig. 1 (q.0, q.1), leading heterogeneous genes including *MUC1*, *KRT7*, *RBM3*, *EPCAM*, *TOMM7* are overall upregulated in cells from the tumor tissue slide. Compared to the total cell population (Fig. 1 (q.0)), a clearer pattern of gene expression shift is demonstrated for 'Epithelial' subtypes (Fig. 1 (q.1), ~20% of the total cells). (After editing) By transforming the sample covariance matrix (SCM) of gene expression collection from one to another, we shift edited gene expression levels from one population towards the compared one (GT). To measure the editing effects on generated (Gen) and reconstructed (Rec) cellular images, we feed edited genes to the GAN (Inversion) models.
Cellular interpretation: We first observe the morphology-level heterogeneity for the total cell population, similar to the gene-level heterogeneity discussed above. However, expected trends of d_{FID} scores immediately arise for the edited epithelial subtypes (Fig. 1 (q.2)), captured by the decreasing d_{FID} between transformed normal epithelial cell population and cancer cells. When focusing on the leading heterogeneous genes *MUC1*, *KRT7*, *RBM3* and *EPCAM*, the editing-driven cellular transition has proven to be effective *w.r.t.* the decreasing d_{FID} between the transformed normal cellular images and the

© The Author 2022. Published by Oxford University Press. All rights reserved. For permissions, please e-mail: journals.permissions@oup.com

tumor counterparts. More importantly, the d_{FID} trends are consistent with the reversed changes of cellular features. This is captured by strongly decreased variance and mildly decreased mean of the nuclear area when shifting the distribution of tumor to normal cellular expression, which confirms effective editing effects achieved by our approach (Fig. 1 (q.3)). As illustrated in the single-cell gallery of Fig. 1 (i.0) and cellular images within the tissue context of Fig. 1 (i.3), we demonstrate the emergence of atypical cellular features for normal epithelial lung cells driven by transforming the expression level of these four leading genes to the malignant spectrum.

References

A. Janesick, R. Shelansky, A. Gottscho, F. Wagner, M. Rouault, G. Beliakoff, M. F. de Oliveira, A. Kohlway, J. Abousoud, C. Morrison, et al. High resolution mapping of the tumor microenvironment using integrated single-cell, spatial and in situ analysis. *Nature Communications* 14.1 (2023): 8353.

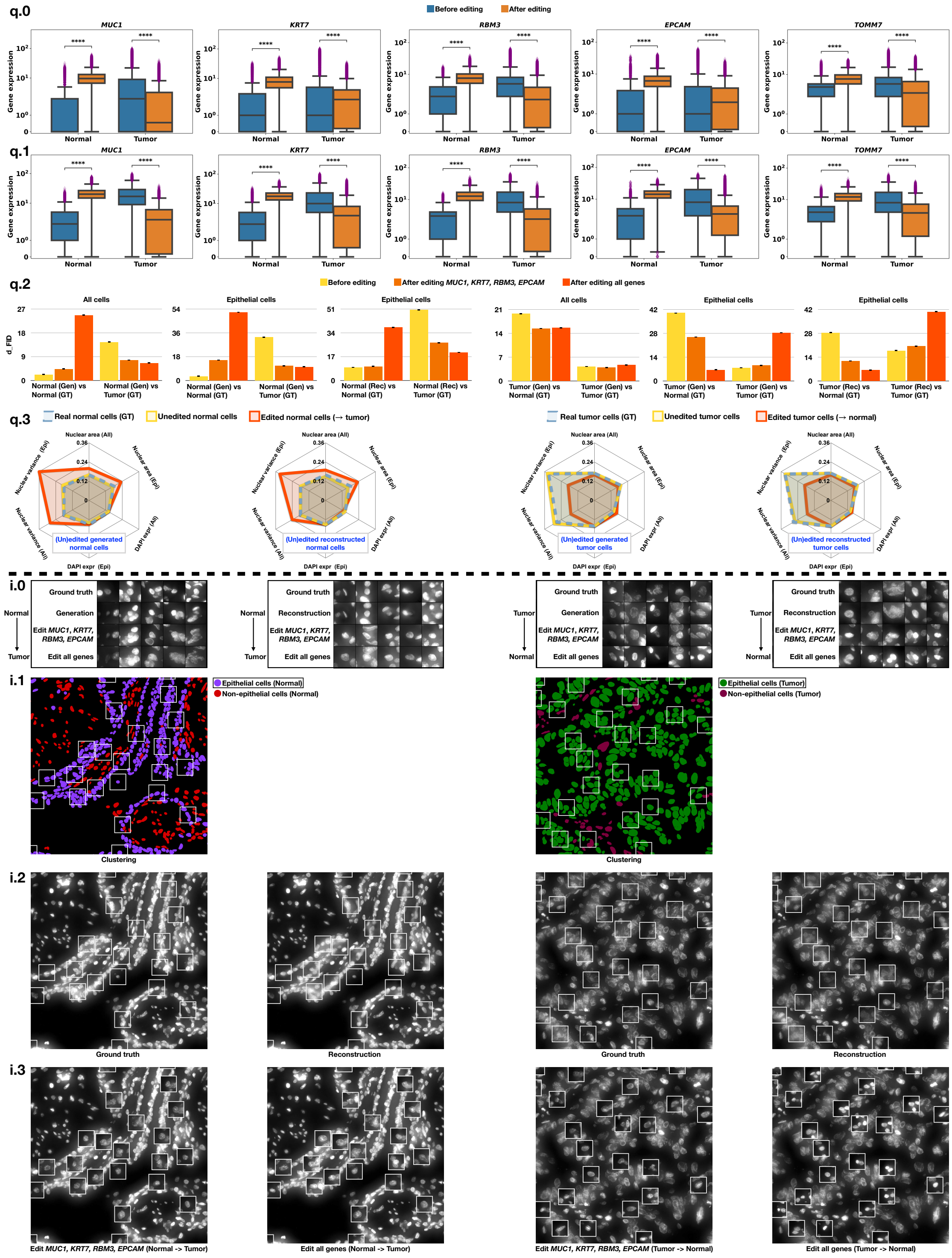

Fig. 1: **q. Numerical quantification of Xenium results**. q.0-q.1. The comparison of gene expression shifts on all the cells (q.0) and the 'Epithelial' (Epi) cells (q.1) of the normal and tumor slide. Here, **** means $p \leq 0.0001$. q.2. For the generated (Gen, GAN) and reconstructed (Rec, GAN Inversion) cells, the d_{FID} comparison of cellular state transitions *w.r.t.* all the cells and Epi subtypes. We randomly repeat the d_{FID} computation four times and report the mean and standard deviation. q.3. The editing effect comparison of interpretable cellular features for all the cells and epithelial cells. **i. Visual interpretation of Xenium results**. i.0. The image gallery of cellular state transitions for Gen and Rec cells. Here, we present the transition that occurred in the DAPI channel. i.1. The visualization of cell subtypes on a region of interest extracted from the normal and tumor slide. i.2. The randomly sampled ground truth and reconstructed cellular images within the bounding boxes. i.3. The morphological transitions of these cellular images guided by edited gene expression levels.

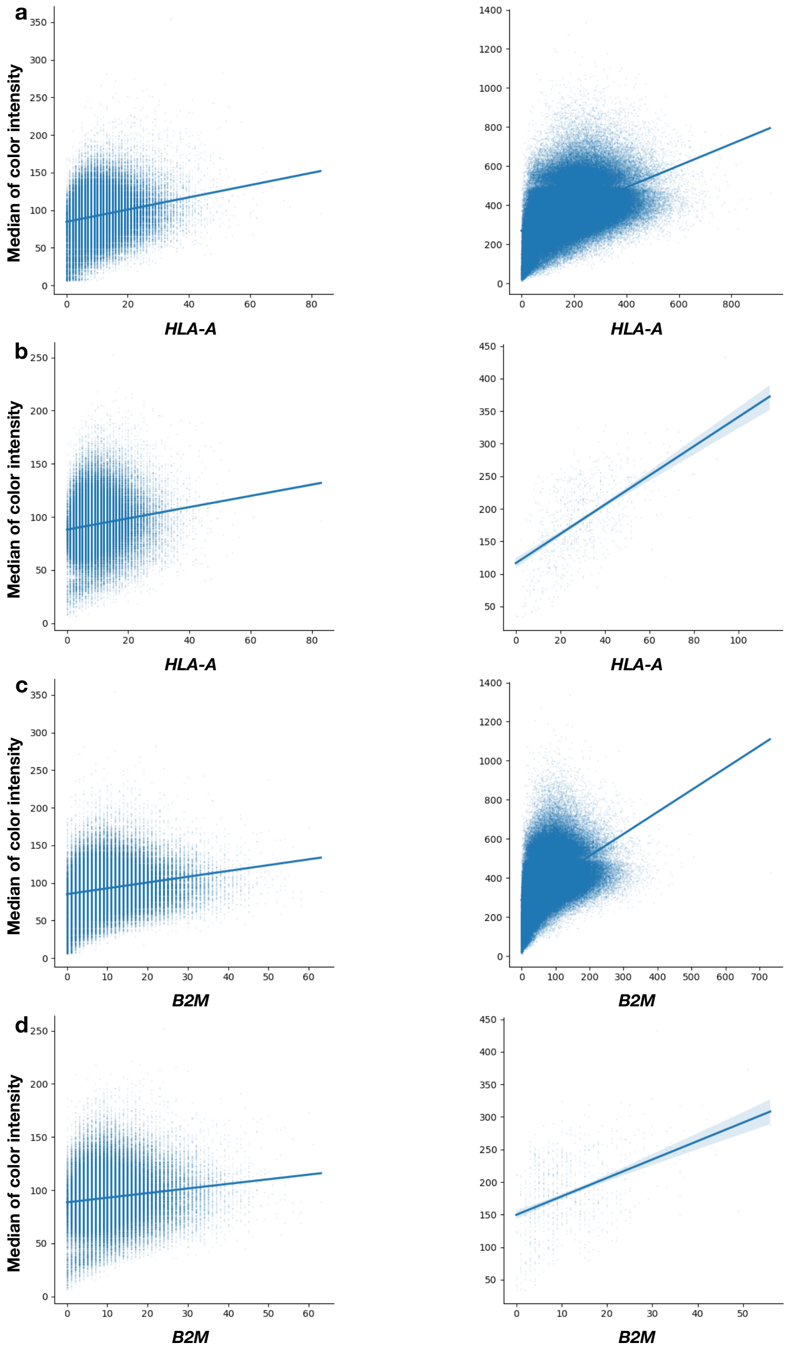

Fig. 2: **The plots of positively correlated median color intensity of CD298/B2M fluorescent marker and *HLA-A*, *B2M* gene expression levels for the CosMx human liver dataset**. **a**. The plots of median color intensity of CD298/B2M and *HLA-A* for all cells from the normal (left) and tumor (right) slide. **b**. The plots of median color intensity of CD298/B2M and *HLA-A* for non-malignant hepatocytes from the normal (left) and tumor (right) slide. **c**. The plots of median color intensity of CD298/B2M and *B2M* for all cells from the normal (left) and tumor (right) slide. **d**. The plots of median color intensity of CD298/B2M and *B2M* for non-malignant hepatocytes from the normal (left) and tumor (right) slide.

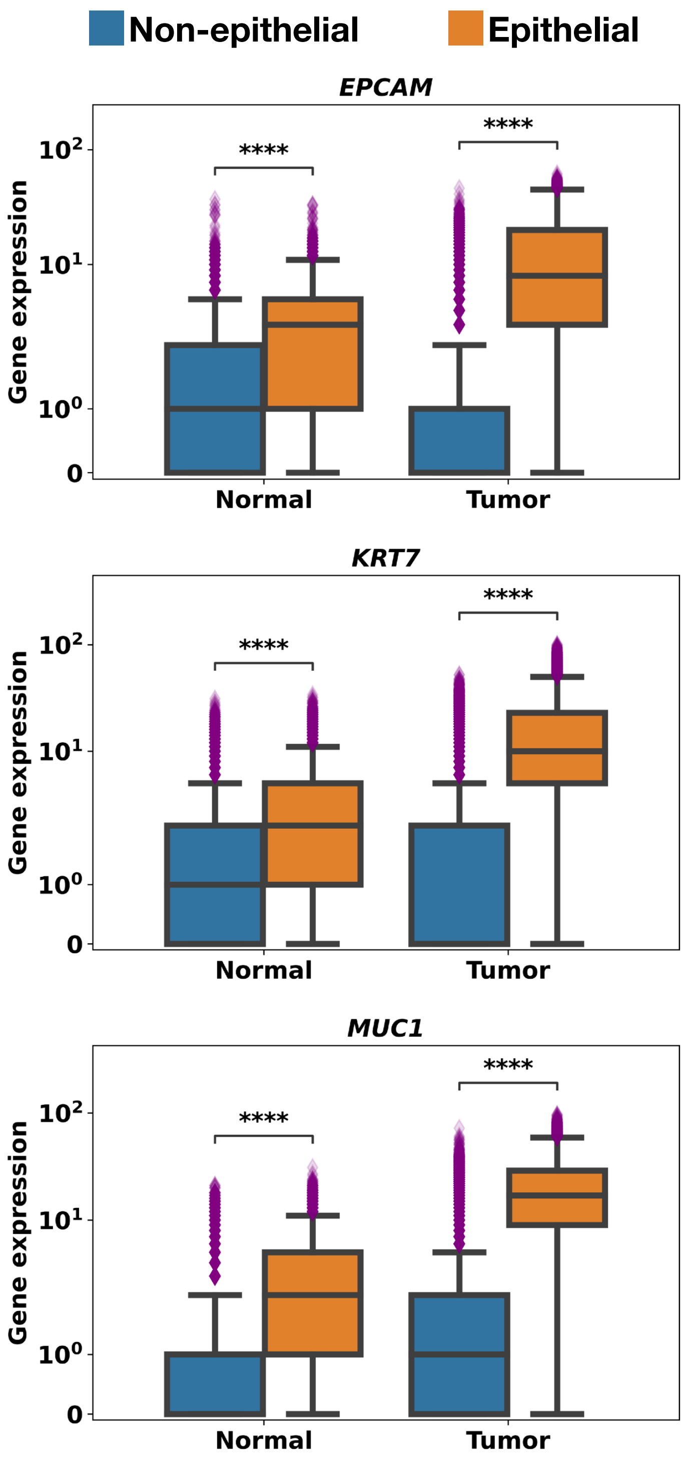

Fig. 3: **The quantitative comparison of the expression level of key genes of interest (*EPCAM* (top), *KRT7* (middle), *MUC1* (bottom)) that stratify the non-epithelial and epithelial-like lung cells of both the normal and tumor slide for the Xenium human lung dataset. Here, **** means p ≤ 0.0001**.

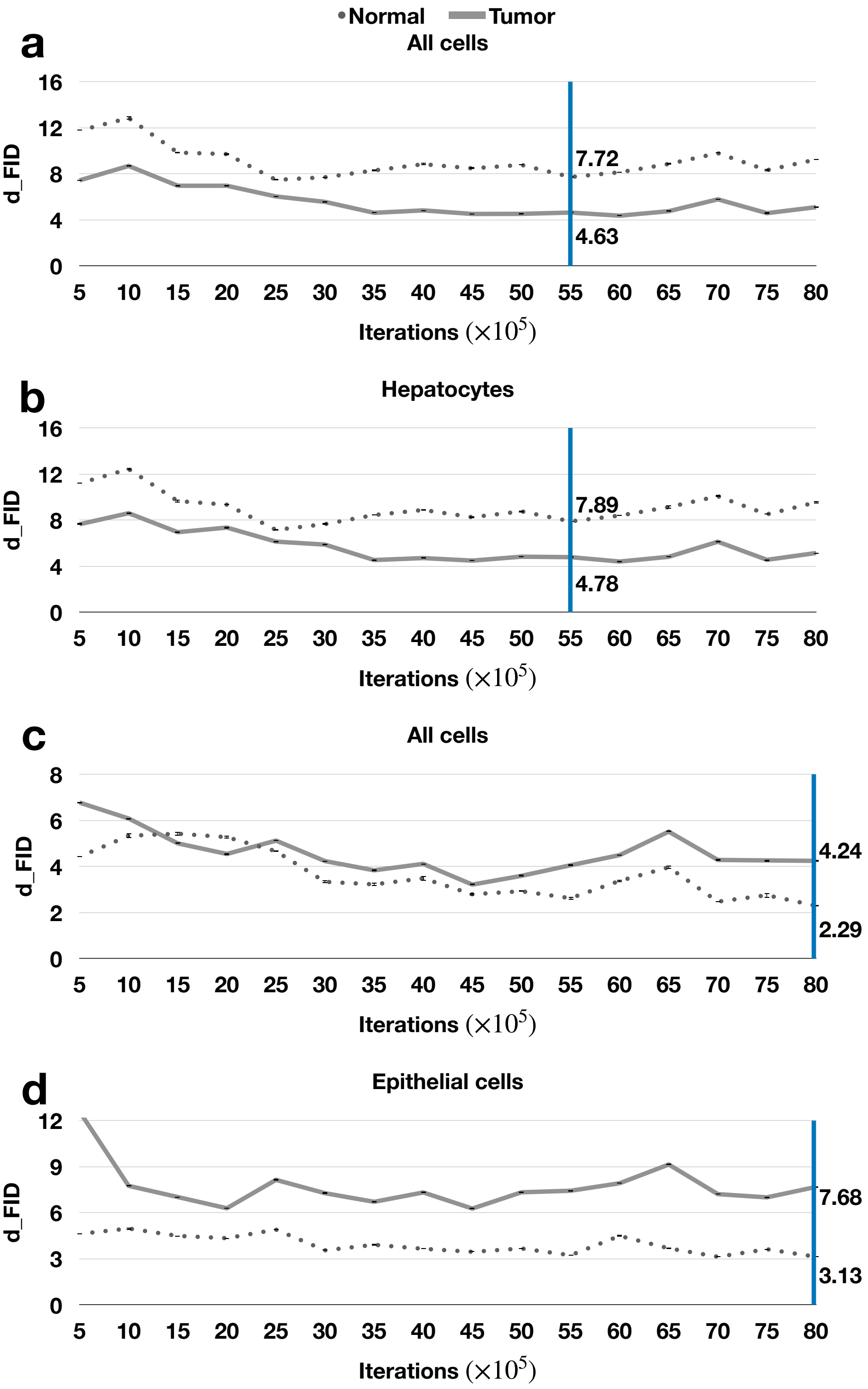

Fig. 4: **The d_{FID} results for GAN training. Here, we randomly repeat the d_{FID} computation four times and report the mean and standard deviation. The overall best d_{FID} scores are highlighted for each case. a**. The d_{FID} curve for all cells from the normal and tumor liver slide of the CosMx dataset. **b**. The d_{FID} curve for all hepatocytes from the normal and tumor liver slide of the CosMx dataset. **c**. The d_{FID} curve for all cells from the normal and tumor lung slide of the Xenium dataset. **d**. The d_{FID} curve for all epithelial cells from the normal and tumor lung slide of the Xenium dataset.

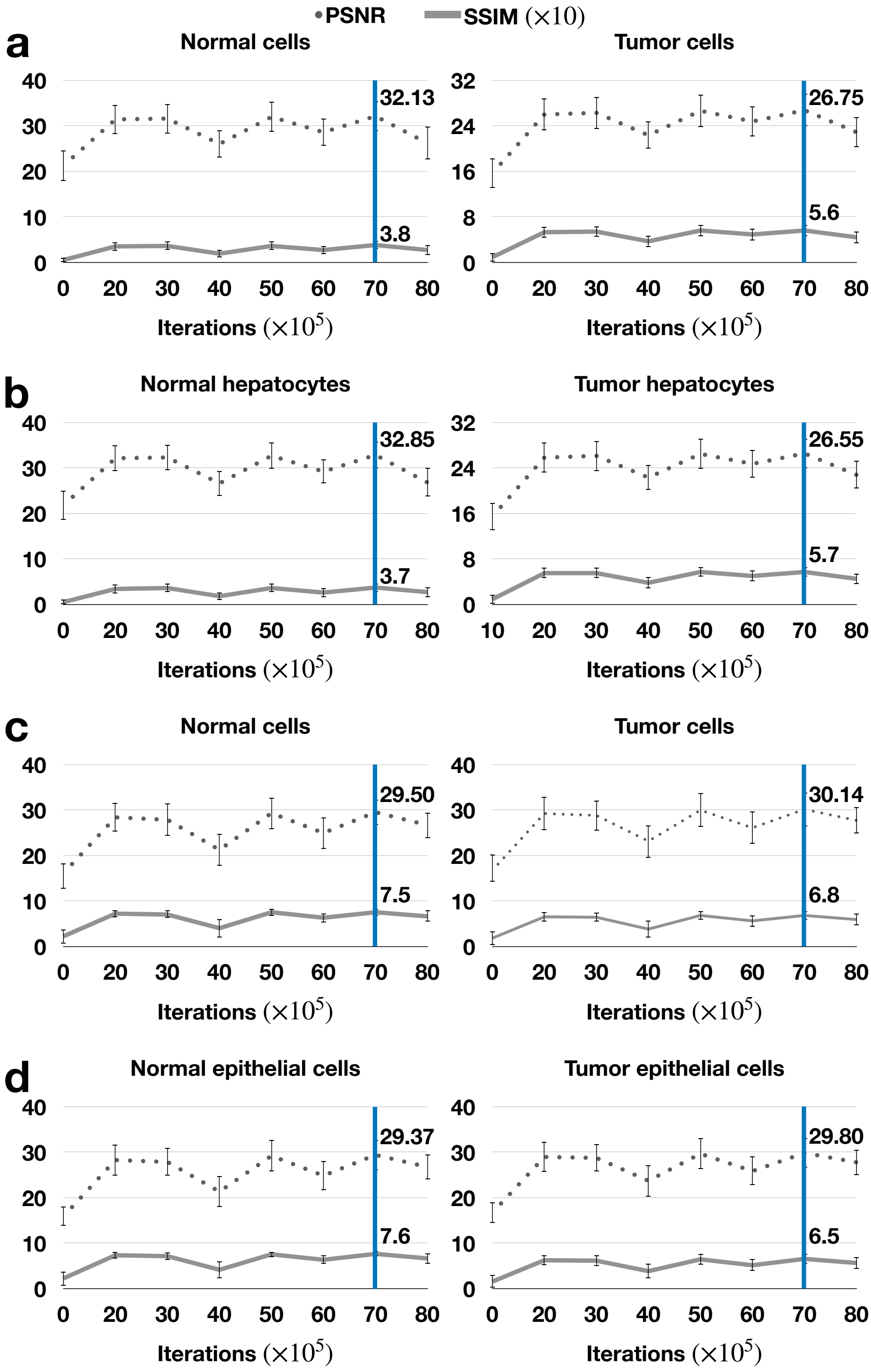

Fig. 5: **The PSNR and SSIM results for GAN Inversion training. Here, we report the mean and standard deviation for both measurements. The overall best PSNR and SSIM scores are highlighted for each case.** **a**. The PSNR and SSIM curve for all cells from the normal and tumor liver slide of the CosMx dataset. **b**. The PSNR and SSIM curve for all hepatocytes from the normal and tumor liver slide of the CosMx dataset. **c**. The PSNR and SSIM curve for all cells from the normal and tumor lung slide of the Xenium dataset. **d**. The PSNR and SSIM curve for all epithelial cells from the normal and tumor lung slide of the Xenium dataset.
